# Outcomes in Patients With Metastatic Renal Cell Carcinoma Who Develop Everolimus-Related Hyperglycemia and Hypercholesterolemia: Combined Subgroup Analyses of the RECORD-1 and REACT Trials

**DOI:** 10.1016/j.clgc.2016.04.011

**Published:** 2016-10

**Authors:** Petri Bono, Stephane Oudard, Istvan Bodrogi, Thomas E. Hutson, Bernard Escudier, Jean-Pascal Machiels, John A. Thompson, Robert A. Figlin, Alain Ravaud, Mert Basaran, Camillo Porta, Sergio Bracarda, Thomas Brechenmacher, Chinjune Lin, Maurizio Voi, Viktor Grunwald, Robert J. Motzer

**Affiliations:** 1 Comprehensive Cancer Center, Helsinki University Hospital and University of Helsinki, Finland; 2 Department of Oncology, Georges Pompidou Hospital, Paris, France; 3 National Institute of Oncology, Budapest, Hungary; 4 Medical Oncology, US Oncology/Baylor-Sammons Cancer Center, Dallas, TX; 5 Immunotherapy Unit, Gustave Roussy Institute, Villejuif, France; 6 Institut Roi Albert II, Service d'Oncologie Médicale, Cliniques universitaires Saint-Luc and Institut de Recherche Clinique et Expérimentale (Pole MIRO), Université catholique de Louvain, Brussels, Belgium; 7 Medical Oncology, Seattle Cancer Care Alliance, Seattle, WA; 8 Samuel Oschin Comprehensive Cancer Institute, Cedars-Sinai Medical Center, Los Angeles, CA; 9 Medical Oncology Hôpital, Saint André CHU, Bordeaux, France; 10 Institute of Oncology, Istanbul University, Istanbul, Turkey; 11 Medical Oncology, IRCCS San Matteo University Hospital Foundation, Pavia, Italy; 12 Medical Oncology, San Donato Hospital, Istituto Toscano Tumori (ITT), Arezzo, Italy; 13 Novartis Pharma S.A.S., Rueil-Malmaison, France; 14 Novartis Pharmaceuticals, East Hanover, NJ; 15 Clinic for Hematology, Hemostasis, Oncology, and Stem Cell Transplantation, Medical School Hannover, Hannover, Germany; 16 Department of Medicine, Genitourinary Oncology Service, Memorial Sloan Kettering Cancer Center, New York, NY

**Keywords:** Class effects of mTOR inhibition, Association of AEs and clinical efficacy, Targeted therapy, mTOR inhibitor

## Abstract

**Background:**

Hyperglycemia and hypercholesterolemia are class effects of mammalian target of rapamycin inhibitors. The purpose of this study was to characterize safety and efficacy of patients with metastatic renal cell carcinoma (mRCC) treated with everolimus in RECORD-1 (REnal Cell cancer treatment with Oral RAD001 given Daily) and REACT (RAD001 Expanded Access Clinical Trial in RCC) who developed these events.

**Patients and Methods:**

Adults with vascular endothelial growth factor–refractory mRCC received everolimus 10 mg/d in the randomized RECORD-1 (n = 277) and open-label REACT (n = 1367) studies. Outcomes included safety, treatment duration, overall response, and progression-free survival for patients who developed hypercholesterolemia or hyperglycemia.

**Results:**

In RECORD-1, 12% (33 of 277) and 20% (55 of 277) of patients developed any grade hyperglycemia or hypercholesterolemia, respectively, with only 6% (78 of 1367) and 1% (14 of 1367) of the same events, respectively, in REACT. Median everolimus treatment duration was similar for patients with hyperglycemia or hypercholesterolemia (RECORD-1, 6.2 and 6.2 months, respectively; REACT, 4.4 and 4.5 months, respectively), but longer than the overall populations (RECORD-1, 4.6 months; REACT, 3.2 months). In RECORD-1/REACT, 82%/68% of patients with hyperglycemia and 75%/71% of patients with hypercholesterolemia achieved partial response or stable disease. The incidence of clinically notable Grade 3 or 4 adverse events, other than anemia and lymphopenia, appeared to be similar across trials and subgroups. Although there was a trend for improved progression-free survival with development of hyperglycemia or hypercholesterolemia, the association was not statistically significant.

**Conclusion:**

Hyperglycemia and hypercholesterolemia were observed in low numbers of patients, and although these events might be associated with improved response to everolimus, the differences were not significant. These findings should be validated with prospective biomarker studies.

## Micro-Abstract

In this study we examined the outcome of metastatic renal cell cancer patients with everolimus treatment-related hyperglycemia and hypercholesterolemia. All patients were treated in 2 large, international prospective trials, RECORD-1 (REnal Cell cancer treatment with Oral RAD001 given Daily) and REACT (RAD001 Expanded Access Clinical Trial in RCC). Patients who experienced these events might have experienced an improved response to everolimus.

## Introduction

Everolimus, a mammalian target of rapamycin (mTOR) inhibitor, has shown efficacy and safety in the treatment of patients with metastatic renal cell carcinoma (mRCC). In the phase III RECORD-1 (REnal Cell cancer treatment with Oral RAD001 given Daily; Clinicaltrials.gov: NCT00410124) study, vascular endothelial growth factor (VEGF)-refractory patients treated with everolimus versus placebo had longer median progression-free survival (PFS; 4.9 months vs. 1.9 months; hazard ratio [HR], 0.33; *P* < .001).^1^ Everolimus was generally well tolerated and there was a low rate of Grade 3 or 4 adverse events (AEs). REACT (RAD001 Expanded Access Clinical Trial in RCC; ClinicalTrials.gov: NCT00655252) was initiated to provide everolimus to patients before it became commercially available and to further assess safety and efficacy of everolimus in VEGF receptor-tyrosine kinase inhibitor–refractory patients with mRCC.^2^ Results of REACT were consistent with those from RECORD-1.

Inhibition of the mTOR pathway provides clinical benefit to patients with mRCC, but the mechanism leads to certain class effects, including hyperglycemia and hyperlipidaemia.^3^ In this analysis, we evaluated the potential association of these AEs with outcomes in patients treated with everolimus in RECORD-1 and REACT.

## Patients and Methods

### Study Design

Study designs for RECORD-1^1^ and REACT^2^ have been previously reported. Both studies included VEGF-refractory patients with mRCC and a Karnofsky performance status (KPS) ≥70% and excluded patients with uncontrolled diabetes as defined according to fasting serum glucose >1.5 times the upper limit of normal (ULN) in RECORD-1 or >2 times the ULN in REACT. In RECORD-1, patients received everolimus 10 mg once daily (n = 277) or placebo (n = 139), both with best supportive care.^1^ In REACT, patients (n = 1367) received everolimus 10 mg once daily until disease progression, unacceptable toxicity, death, discontinuation (patient or physician discretion), commercial availability, or June 15, 2010 (whichever came first).^2^ In both studies, dose reduction to 5 mg daily was permitted if unacceptable toxicity occurred.

### Assessments

Safety was assessed at baseline, then monthly for up to 28 days after the last dose of study drug and included physical examination, assessment of KPS, electrocardiography, and hematology, chemistry, lipid, and coagulation profiles. AEs and laboratory abnormalities were graded according to the National Cancer Institute's Common Terminology Criteria for AEs (version 3.0). In RECORD-1, all AEs were monitored and recorded. Fasting glucose and total cholesterol levels were measured at screening and on day 1 of each treatment cycle and at study discontinuation. If an increase in serum glucose or cholesterol required dose modification or interruption, testing was repeated until levels returned to baseline. In REACT, data on all Grade 3 and 4 AEs were collected; data on Grades 1 and 2 AEs were collected only if their occurrence resulted in a change in study drug administration (dose modification/interruption or treatment discontinuation).

In RECORD-1, tumor measurements were assessed (Response Evaluation Criteria In Solid Tumors [RECIST] version 1.0) at screening, every 8 weeks thereafter, and at study discontinuation. Assessments were done by a local investigator and central review. In REACT, best overall tumor response was assessed (RECIST version 1.0) at baseline, every 3 months for the first year, every 6 months thereafter, and at study discontinuation. Assessment was done by local investigator review.

Patients included in this subgroup analysis developed hyperglycemia or hypercholesterolemia during everolimus treatment. In RECORD-1, patients who developed any grade hyperglycemia or hypercholesterolemia were included. In REACT, patients who developed Grades 3 or 4 hyperglycemia or hypercholesterolemia and patients who developed Grades 1 or 2 hyperglycemia or hypercholesterolemia that resulted in a change in study drug administration were included. Descriptive statistics were used. Logistic regression analysis was performed to assess the effect of elevated baseline glucose or cholesterol levels on best overall tumor response. A landmark analysis of PFS according to central assessment was performed for patients treated for ≥8 weeks and according to occurrence of any grade hyperglycemia or hypercholesterolemia within the first 8 weeks of treatment. The landmark analysis was used to correct bias inherent to the confounding effect of duration of exposure and the 8-week cutoff was chosen because it corresponded to the first scheduled tumor assessment, plus a high number of hyperglycemia or hypercholesterolemia events occurred by this time. PFS estimates were obtained using the Kaplan–Meier method. HRs were calculated using the Cox proportional hazards model. Statistical analyses were performed using SAS version 9.3 (SAS Institute Inc, Cary, NC).

## Results

### Patients

In RECORD-1, 12% of patients (33 of 277) developed hyperglycemia and 20% of patients (55 of 277) developed hypercholesterolemia; there was a significant correlation between development of the 2 events (χ^2^ test, *P* = .0005). Among the 33 cases of hyperglycemia, 7 were Grade 1 (21%), 9 were Grade 2 (27%), and 17 were Grade 3 (52%). Among the 55 cases of hypercholesterolemia, 30 were Grade 1 (55%), 16 were Grade 2 (29%), and 9 were Grade 3 (16%). In REACT, 6% of patients (78 of 1367) developed hyperglycemia and 1% of patients (14 of 1367) developed hypercholesterolemia. Among the 78 cases of hyperglycemia, 3 were Grade 1 or 2 (4%), 67 were Grade 3 (86%), and 8 were Grade 4 (10%), and among the 14 cases of hypercholesterolemia, 2 were Grade 1 or 2 (14%), 9 were Grade 3 (64%), and 3 were Grade 4 (21%).

Baseline characteristics (Table 1) were similar between patients who developed either AE and all everolimus-treated patients. One notable exception was that in both trials, a greater proportion of Asian patients appeared more likely to develop hyperglycemia. Although representing 6% of the RECORD-1 population and 8% of the REACT population, Asian patients represented 12% and 17% of patients with hyperglycemia from RECORD-1 or REACT, respectively.

**Figure 1:**
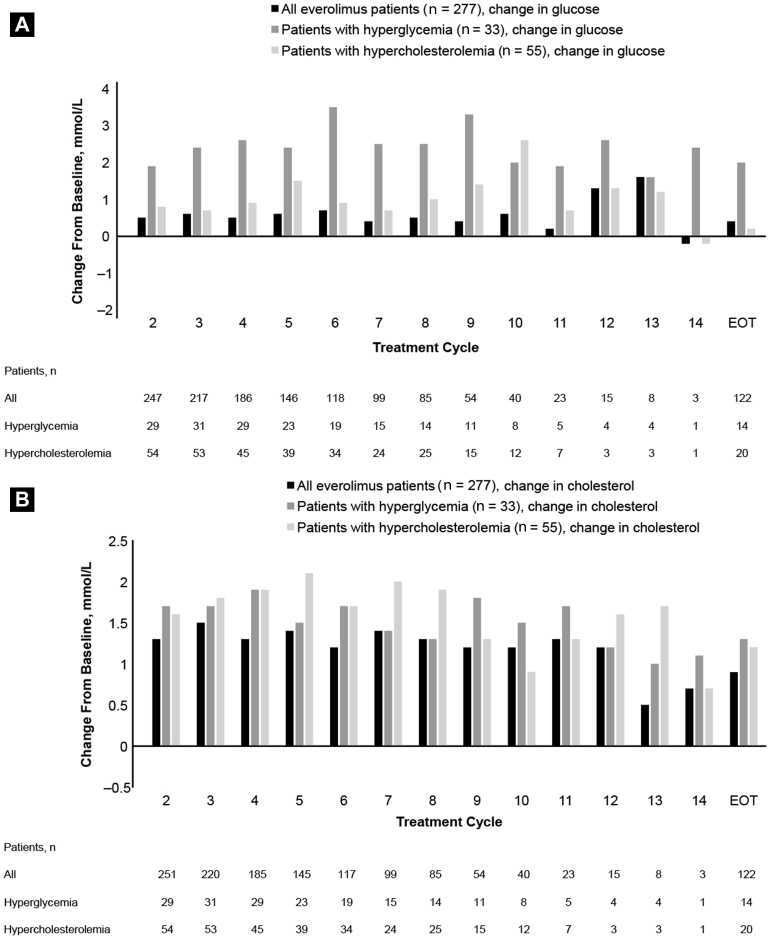
Median Changes From Baseline Over Time in (**A**) Glucose and (**B**) Cholesterol Levels for Patients Treated With Everolimus in RECORD-1 (REnal Cell Cancer Treatment With Oral RAD001 Given Daily) Abbreviation: EOT = end of treatment.

Among RECORD-1 patients who developed hyperglycemia, median baseline glucose was 6.3 mmol/L (range, 5-11) and the median increase in serum glucose was 2.0 mmol/L at end of treatment. Among RECORD-1 patients who developed hypercholesterolemia, median baseline cholesterol was 4.9 mmol/L (range, 3-9) and the median increase in serum cholesterol was 1.2 mmol/L at end of treatment. Increases over time in glucose levels of patients with hyperglycemia and in cholesterol levels of patients with hypercholesterolemia are shown in Figure 1. In RECORD-1, 63% of patients with hyperglycemia were taking medication to control their diabetes at the start of the study (metformin, 18%; sulfonamide urea, 15%; and insulin, 30%). In addition, 15% of patients with hyperglycemia were taking lipid-lowering medication (hydroxymethyl gluctaryl coenzyme A [HMG CoA] reductase inhibitors) at the start of the study. Among patients with hypercholesterolemia, 22% (12 of 55) were taking lipid-lowering medication (HMG CoA reductase inhibitors) and 22% (12 of 55) were taking medication to control diabetes (metformin, 4%; sulfonamide urea, 4%; and insulin, 14%) at the start of the study. REACT patients with hyperglycemia were taking metformin (31%; 24 of 78), sulfonamide urea (27%; 21 of 78), and/or insulin (54%; 42 of 78) for glucose control at the start of the study. In addition, 44% of patients with hyperglycemia were taking lipid-lowering medication at the start of the study (HMG CoA reductase inhibitors alone or in combination with other lipid-lowering drugs). Among patients with hypercholesterolemia, 29% (4 of 14) were taking HMG CoA reductase inhibitors alone or in combination with other lipid modifiers at the start of the study (1 patient was taking sulfonamide urea).

### Dose Modifications and Treatment Duration

In RECORD-1, 4.3 weeks was the median time to development of hyperglycemia (n = 33; 95% confidence interval [CI], 4.0-8.0) or hypercholesterolemia (n = 55; 95% CI, 4.1-8.0). Among RECORD-1 patients who developed hyperglycemia, 6% (2 of 33) required a dose adjustment or study drug interruption and 61% (20 of 33) received concomitant medications to manage the event. No patients discontinued treatment because of hyperglycemia. Among RECORD-1 patients who developed hypercholesterolemia, 2% (1 of 55) required a dose adjustment or study drug interruption and 31% (17 of 55) received concomitant medications to manage the event. No patients discontinued treatment because of hypercholesterolemia.

Among REACT patients who developed hyperglycemia, 36% (28 of 78) required a dose adjustment (24 [31%] Grade 3 and 3 [4%] Grade 4) and 68% (53 of 78) received concomitant medications (43 [55%] Grade 3 and 7 [9%] Grade 4). Among patients who developed hypercholesterolemia, 29% (4 of 14) required a dose adjustment (1 [7%] Grade 3 and 1 [7%] Grade 4), and 43% (6 of 14) received concomitant medications (3 [21%] Grade 3 and 2 [14%] Grade 4).

In REACT, 4% of patients discontinued because of hyperglycemia (0 Grade 3 and 2 [3%] Grade 4) and 7% of patients discontinued because of hypercholesterolemia (1 [7%] Grade 3). RECORD-1 and REACT patients who developed hyperglycemia or hypercholesterolemia had longer median treatment duration than the total study populations. In RECORD-1, median treatment duration was 6.2 months each for patients with either AE and 4.6 months for all everolimus-treated patients. In REACT, median treatment duration was 4.4 months for patients with hyperglycemia, 4.5 months for patients with hypercholesterolemia, and 3.2 months for the total population.

**Figure 2:**
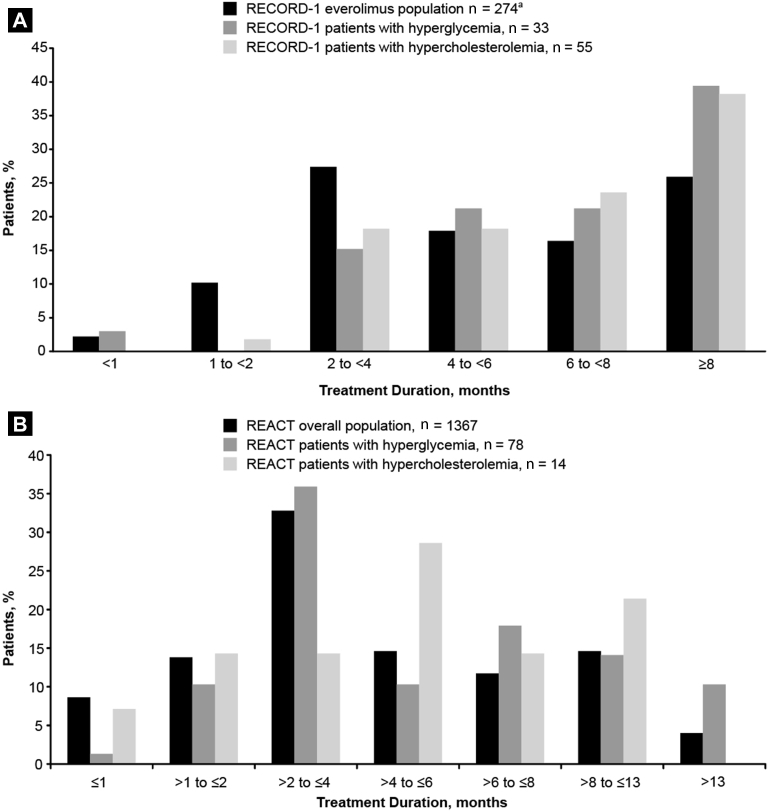
Everolimus Treatment Duration in (**A**) RECORD-1 (REnal Cell Cancer Treatment With Oral RAD001 Given Daily) and (**B**) REACT (RAD001 Expanded Access Clinical Trial in RCC) for the Overall Populations and for Patients With Hyperglycemia or Hypercholesterolemia. ^a^Safety Population (n = 274); 3 Patients Who Were Randomized Were Excluded From the Safety Population

In RECORD-1 and REACT, a greater percentage of patients with either AE than of patients in the total study populations had a treatment duration of ≥8 months (Figure 2). In RECORD-1, 39% of patients with hyperglycemia, 38% of patients with hypercholesterolemia, and 26% of the everolimus-treated population were treated for ≥8 months. In REACT, 24% of patients with hyperglycemia, 21% of patients with hypercholesterolemia, and 19% of the total population were treated for >8 months.

### Efficacy

Similar percentages of patients in RECORD-1 and REACT who developed either AE achieved partial response (PR) or stable disease (SD) from treatment with everolimus (Table 2). In RECORD-1 and REACT, 82% and 68% of patients with hyperglycemia, respectively, achieved PR or SD. Similarly, in RECORD-1 and REACT, 75% and 71% of patients with hypercholesterolemia, respectively, achieved PR or SD. In all RECORD-1 and REACT patients, 69% and 54%, respectively, achieved PR or SD. In RECORD-1, patients with elevated baseline glucose levels had a greater tumor response rate than patients without elevated baseline glucose level (odds ratio, 1.33; 95% CI, 1.06-1.67).

In RECORD-1, there was no significant effect of elevated baseline glucose level on PFS; however, patients with elevated baseline cholesterol level had a greater chance of experiencing shorter PFS than patients without elevated baseline cholesterol level (HR, 0.83; 95% CI, 0.75-0.93). In the RECORD-1 landmark analysis at week 8, median PFS was 5.9 months (95% CI, 4.0-not estimable [NE]) and 5.4 months (95% CI, 4.3-5.6) for those who did and did not develop hyperglycemia, respectively (HR, 0.70; 95% CI, 0.37-1.30) and 5.9 months (95% CI, 4.0-NE) and 5.1 months (95% CI, 4.3-5.6) for those who did and did not develop hypercholesterolemia, respectively (HR, 0.76; 95% CI, 0.45-1.27; Table 3). At the 8-week landmark, 87% of patients (240 of 277) had been treated with everolimus for ≥8 weeks. Although there was a trend for an association between development of these AEs and longer PFS, it was not statistically significant. Of note, the same analysis was conducted without adjustment (no landmark; Table 3). Although results of this unadjusted analysis were statistically significant, they should be interpreted with caution because of the confounding effect of duration of exposure.

### Safety

In RECORD-1 and REACT, the incidence of patients who experienced Grade 3 or 4 anemia was higher for patients with hyperglycemia and lower for patients with hypercholesterolemia, compared with patients in the total study populations (Table 4). In RECORD-1, the incidence of patients who experienced Grade 3 or 4 lymphopenia was higher for patients with hypercholesterolemia and lower for patients with hyperglycemia, compared with patients in the total study population. In REACT, more patients with hyperglycemia or hypercholesterolemia than patients in the overall population experienced Grade 3 or 4 lymphopenia. The incidence of other clinically notable Grade 3 or 4 AEs appeared to be similar across trials and subgroups (Table 4).

## Discussion

The correlation of specific AEs with clinical efficacy of targeted therapies in mRCC has been controversial. Hypertension is a class effect of anti-VEGF therapy.^4^, ^5^, ^6^, ^7^ Results of some studies have shown that treatment-induced hypertension was associated with improved outcomes in patients treated with axitinib,^8^, ^9^, ^10^, ^11^ bevacizumab,^12^ sorafenib,^13^ and sunitinib.^12^, ^14^, ^15^ However, results of another study showed a weak correlation between axitinib steady-state exposure and change in diastolic blood pressure from baseline.^16^ Hyperglycemia and hyperlipidaemia are class effects of mTOR inhibition.^3^ Results of a retrospective study of patients from the temsirolimus registration trial showed that temsirolimus' effect on cholesterol, but not on glucose, predicted its effect on survival; longer survival with temsirolimus was observed in patients with larger increases in cholesterol.^17^ We found that everolimus-treated patients from RECORD-1 and REACT who developed hyperglycemia or hypercholesterolemia had longer median treatment durations than patients in the total populations. A higher percentage of patients who developed hyperglycemia or hypercholesterolemia than in the total RECORD-1 and REACT populations achieved PR or SD. In this analysis of RECORD-1, elevated baseline glucose level was associated with an increased rate of tumor response. Although the 8-week landmark analysis showed a trend for improved PFS and development of hyperglycemia or hypercholesterolemia, the associations were not statistically significant. Results of the landmark analysis should be interpreted with caution because of low patient numbers and low numbers of PFS events.

In RECORD-1, abnormal serum glucose values were observed in 57% of patients who received everolimus and 25% of patients who received placebo; the incidence of hyperglycemia was 8% and <1%, respectively.^1^, ^18^ Most cases of hyperglycemia occurred in patients with abnormal fasting glucose levels before treatment; 1 new case of diabetes mellitus was reported during the trial.^18^ Among patients who developed hyperglycemia in RECORD-1 and REACT, 52% and 96%, respectively, experienced it as a Grade 3 or 4 event. Among patients who developed hypercholesterolemia in RECORD-1 and REACT, 16% and 86%, respectively, experienced it as a Grade 3 or 4 event. In REACT, data on Grades 1 and 2 AEs were captured only if they resulted in a change in study drug administration (ie, dose modification, temporary interruption, or treatment discontinuation). This might explain the higher percentage of patients in the REACT hyperglycemia and hypercholesterolemia subgroups who experienced Grade 3 or 4 events compared with the corresponding groups from RECORD-1. It might also explain the smaller sizes of the REACT subgroups relative to the total study population than the corresponding subgroups from RECORD-1. Although Asian patients treated with everolimus in both studies appeared more likely to develop hyperglycemia, patients from European countries who participated in REACT developed the event at rates similar to the overall population.^19^ Management guideline goals for patients with cancer who develop mTOR inhibitor-associated hyperlipidaemia (elevations in total cholesterol, low-density lipoprotein, and triglyceride levels) or hyperglycemia are to decrease short-term morbidity associated with these AEs.^20^ All patients who receive mTOR inhibitor therapy should be screened and regularly monitored for hyperlipidemia and hyperglycemia.

## Conclusion

These results suggest that development of hyperglycemia and hypercholesterolemia occur in low numbers of patients and do not lead to everolimus discontinuation. Most patients who developed these AEs continued therapy for a longer duration than patients in the overall population. Patients who experience these events might also experience an improved response to everolimus. There was a trend for an association between development of hyperglycemia and hypercholesterolemia and longer PFS, but the differences were not statistically significant.

### Clinical Practice Points

• Hyperglycemia and hypercholesterolemia are class effects of mTOR inhibitors such as everolimus.
• In this analysis, we evaluated the potential association of these AEs with outcomes in patients treated with everolimus in RECORD-1 and REACT.
• Hyperglycemia and hypercholesterolemia were observed in low numbers of patients, and although these events might be associated with improved response to everolimus, the differences were not statistically significant.
• These findings should be validated with prospective biomarker studies.

## Disclosure

P.B. received compensation from Novartis, Pfizer, Bristol-Myers Squibb, and Merck Sharp & Dohme and a research grant from Novartis. S.O. received advisory compensation from Bayer, Pfizer, Novartis, GlaxoSmithKline, and Bristol-Myers Squibb. T.E.H. received advisory compensation and research grants from Pfizer, Novartis, Exelexis, Johnson, and Astellas, and honoraria from Pfizer, Novartis, Johnson, and Astellas. B.E. received advisory compensation from Novartis, Bayer, GlaxoSmithKline, and Pfizer. J.-P.M. received advisory compensation from Merck Sharp & Dohme and research grants from Novartis, Janssen, and Bayer, and is a board member at Boehringer. R.A.F. received a research grant from GlaxoSmithKline. A.R. received advisory compensation from Pfizer, Novartis, GlaxoSmithKline, and Roche; reimbursement for transportation and housing for meetings and speeches from Pfizer, Novartis, GlaxoSmithKline, Roche, and Merck Sharp & Dohme; and research grants from Pfizer and Novartis. M.B. received advisory compensation from Sanofi. C.P. received a research grant from Pfizer and honoraria from Pfizer, Novartis, GlaxoSmithKline, Bayer-Schering, Bristol-Myers Squibb, Astellas, and Pierre Fabre. S.B. received advisory compensation from Pfizer, Novartis, Bayer, Janssen, and Astellas. T.B., C.L., and M.V. are Novartis employees. V.G. received advisory compensation from Bristol-Myers Squibb, Novartis, Pfizer, and Bayer. R.J.M. received grants and personal fees from Novartis, Pfizer, and GlaxoSmithKline, grants from Genentech and GlaxoSmithKline, trial support paid to employer by Novartis, Genentech, and GlaxoSmithKline, and advisory compensation from Novartis and Pfizer. I.B. and J.A.T. have stated that they have no conflicts of interest.

## Figures and Tables

**Table 1:** Baseline Characteristics of All Patients in the Everolimus Arm of RECORD-1, All Patients in REACT, and the Subgroups of Everolimus-Treated Patients in Each Trial Who Developed Hyperglycemia or Hypercholesterolemia

Characteristic	RECORD-1 Everolimus Arm			REACT		
	All Patients (n = 277)	Patients With Hyperglycemia (n = 33)	Patients With Hypercholesterolemia (n = 55)	All Patients (n = 1367)	Patients With Hyperglycemia (n = 78)	Patients With Hypercholesterolemia (n = 14)
**Median Age, Years**	61	62	61	63	63	62
**Sex, n (%)**						
Male	216 (78)	26 (798)	44 (80)	989 (72)	54 (69)	9 (64)
Female	61 (22)	7 (21)	11 (20)	378 (287)	24 (31)	5 (36)
**Race, n (%)**						
White	246 (89)	28 (85)	51 (93)	1220 (89)	64 (82)	11 (79)
Asian	16 (6)	4 (12)	3 (6)	114 (8)	13 (17)	1 (7)
Other	11 (4)	1 (3)	1 (2)	33 (2)	1 (1)	2 (14)
Missing	4 (1)	0	0	0	0	0
**Tumor Histology, n (%)**						
Clear cell adenocarcinoma	266 (96)	32 (97)	53 (96)	1283 (94)	73 (94)	14 (100)
Other	11 (4)	1 (3)	2 (4)	75 (6)	5 (6)	0
Missing	0	0	0	9 (1)	0	0
**Histological Grade, n (%)**						
Well differentiated	22 (8)	1 (3)	6 (11)	76 (6)	3 (4)	0
Moderately differentiated	57 (21)	10 (30)	14 (26)	369 (27)	18 (23)	5 (36)
Poorly differentiated	84 (30)	10 (30)	17 (31)	351 (26)	21 (27)	3 (21)
Undifferentiated	17 (6)	3 (9)	1 (2)	106 (8)	5 (6)	1 (7)
Unknown	97 (35)	9 (27)	17 (31)	465 (34)	31 (40)	5 (36)
**Median Cholesterol, mmol/L (range)**^a^	4.6 (2-10)	4.9 (3-8)	4.9 (3-9)	–	–	–
**Median Glucose, mmol/L (range)**^b^	5.5 (3-19)	6.3 (5-11)	5.4 (4-11)	–	–	–

Abbreviations: REACT = RAD001 Expanded Access Clinical Trial in RCC; RECORD-1 = REnal Cell cancer treatment with Oral RAD001 given Daily.

a Normal level of total cholesterol is < 5.2 mmol/L (<201 mg/dL).

b Normal range of glucose is 4.5-7.0 mmol/L (81-126 mg/dL).

**Table 2:** Best Overall Response to Everolimus in RECORD-1 and REACT for the Overall Populations and for Patients Who Developed Hyperglycemia or Hypercholesterolemia

Best Overall Response, n (%)	RECORD-1 Everolimus Arm			REACT		
	All Patients (n = 277)	Patients With Hyperglycemia (n = 33)	Patients With Hypercholesterolemia (n = 55)	All Patients (n = 1364)	Patients With Hyperglycemia (n = 78)	Patients With Hypercholesterolemia (n = 14)
**Partial Response**	5 (2)	2 (6)	2 (4)	23 (2)	2 (5)	0
**Stable Disease**	185 (67)	25 (76)	39 (71)	705 (52)	51 (65)	10 (71)
**Progressive Disease**	57 (21)	4 (12)	10 (18)	324 (24)	13 (17)	1 (7)
**Unknown**^a^	30 (11)	2 (6)	4 (7)	315 (23)	12 (15)	3 (21)

Abbreviations: REACT = RAD001 Expanded Access Clinical Trial in RCC; RECORD-1 = Renal Cell cancer treatment with Oral RAD001 given Daily.

a Cases not qualifying for confirmed complete response or partial response and without stable disease after >6 weeks or early progression of disease within the first 12 weeks.

**Table 3:** RECORD-1: Analysis of Progression-Free Survival (Central Review) and Occurrence of Hyperglycemia or Hypercholesterolemia During the Entire Study (No Landmark) and Within the First 8 Weeks of Treatment (Landmark)

	No Landmark	8 Weeks
**Patients Treated for At Least 8 Weeks, n/Total n (%)**	277/277 (100)	240/277 (87)
**Hyperglycemia/No Hyperglycemia**		
Patients, n/n	33/244	21/219
PFS events, n/n	15/140	11/123
Median PFS (95% CI), months	7.3 (4.0-NE)/4.5 (3.8-5.5)	5.9 (4.0-NE)/5.4 (4.3-5.6)
HR (95% CI)	0.50 (0.29-0.85)	0.70 (0.37-1.30)
**Hypercholesterolemia/No Hypercholesterolemia**		
Patients, n/n	55/222	35/205
PFS events, n/n	28/127	17/117
Median PFS (95% CI), months	7.3 (5.2-11.9)/4.3 (3.7-5.4)	5.9 (4.0-NE)/5.1 (4.3-5.6)
HR (95% CI)	0.61 (0.40-0.92)	0.76 (0.45-1.27)

Abbreviations: HR = hazard ratio; NE = not estimable; PFS = progression-free survival; RECORD-1 = REnal Cell cancer treatment with Oral RAD001 given Daily.

**Table 4:** Combined Grades 3 and 4 Clinically Notable Adverse Events in RECORD-1 and REACT Patients Who Developed Hyperglycemia or Hypercholesterolemia^a^

Adverse Event	RECORD-1 Everolimus Arm			
	All Patients (n = 274)^b^	Patients With Hyperglycemia (n = 33)	Patients With Hypercholesterolemia (n = 55)	Patients With Hyperglycemia and Hypercholesterolemia (n = 15)
**Anemia**	28 (10)	5 (15)	2 (4)	1 (7)
**Hyperglycemia**	17 (6)	17 (52)	10 (18)	10 (67)
**Lymphopenia**	12 (4)	1 (3)	5 (9)	1 (7)
**Stomatitis**	12 (4)	1 (3)	3 (6)	0
**Hypercholesterolemia**	9 (3)	3 (9)	9 (16)	4 (27)
**Pneumonitis**	7 (3)	0	0	0
	**REACT**			
	**All Patients (n = 1364)**	**Patients With Hyperglycemia (n = 78)**	**Patients With Hypercholesterolemia (n = 14)**	

**Anemia**	183 (13)	21 (27)	1 (7)	
**Hyperglycemia**	75 (6)	75 (96)	0	
**Stomatitis**	74 (5)	7 (9)	0	
**Pneumonitis**	37 (3)	3 (4)	0	
**Hypercholesterolemia**	12 (<1)	0	12 (86)	
**Lymphopenia**	12 (<1)	2 (3)	1 (7)	

Data are presented as n (%).

Abbreviations: REACT = RAD001 Expanded Access Clinical Trial in RCC; RECORD-1 = REnal Cell cancer treatment with Oral RAD001 given Daily.

a Clinically notable adverse events with a combined Grade 3/4 incidence of >2% in either the total RECORD-1 population or the total REACT population are shown.

b Safety population (n = 274); 3 patients who were randomized were excluded from the safety population.
